# SUCNR1-mediated chemotaxis of macrophages aggravates obesity-induced inflammation and diabetes

**DOI:** 10.1007/s00125-017-4261-z

**Published:** 2017-04-05

**Authors:** Janna A. van Diepen, Joris H. Robben, Guido J. Hooiveld, Claudia Carmone, Mohammad Alsady, Lily Boutens, Melissa Bekkenkamp-Grovenstein, Anneke Hijmans, Udo F. H. Engelke, Ron A. Wevers, Mihai G. Netea, Cees J. Tack, Rinke Stienstra, Peter M. T. Deen

**Affiliations:** 1 Department of Internal Medicine, Radboud University Medical Center, Nijmegen, the Netherlands; 2 Department of Physiology, Radboud University Medical Center, PO Box 9101, 6500 HB Nijmegen, the Netherlands; 3 Nutrition, Metabolism and Genomics Group, Division of Human Nutrition, Wageningen University, Wageningen, the Netherlands; 4 Department of Laboratory Medicine, Radboud University Medical Center, Nijmegen, the Netherlands

**Keywords:** Adipose tissue, Chemotaxis, Glucose, Inflammation, Macrophage, Obesity, Succinate, TCA cycle

## Abstract

**Aims/hypothesis:**

Obesity induces macrophages to drive inflammation in adipose tissue, a crucial step towards the development of type 2 diabetes. The tricarboxylic acid (TCA) cycle intermediate succinate is released from cells under metabolic stress and has recently emerged as a metabolic signal induced by proinflammatory stimuli. We therefore investigated whether succinate receptor 1 (SUCNR1) could play a role in the development of adipose tissue inflammation and type 2 diabetes.

**Methods:**

Succinate levels were determined in human plasma samples from individuals with type 2 diabetes and non-diabetic participants. Succinate release from adipose tissue explants was studied. *Sucnr1* ^*−*/−^ and wild-type (WT) littermate mice were fed a high-fat diet (HFD) or low-fat diet (LFD) for 16 weeks. Serum metabolic variables, adipose tissue inflammation, macrophage migration and glucose tolerance were determined.

**Results:**

We show that hypoxia and hyperglycaemia independently drive the release of succinate from mouse adipose tissue (17-fold and up to 18-fold, respectively) and that plasma levels of succinate were higher in participants with type 2 diabetes compared with non-diabetic individuals (+53%; *p* < 0.01). *Sucnr1* ^*−*/−^ mice had significantly reduced numbers of macrophages (0.56 ± 0.07 vs 0.92 ± 0.15 F4/80 cells/adipocytes, *p* < 0.05) and crown-like structures (0.06 ± 0.02 vs 0.14 ± 0.02, CLS/adipocytes *p* < 0.01) in adipose tissue and significantly improved glucose tolerance (*p* < 0.001) compared with WT mice fed an HFD, despite similarly increased body weights. Consistently, macrophages from *Sucnr1* ^*−*/−^ mice showed reduced chemotaxis towards medium collected from apoptotic and hypoxic adipocytes (−59%; *p* < 0.05).

**Conclusions/interpretation:**

Our results reveal that activation of SUCNR1 in macrophages is important for both infiltration and inflammation of adipose tissue in obesity, and suggest that SUCNR1 is a promising therapeutic target in obesity-induced type 2 diabetes.

**Data availability:**

The dataset generated and analysed during the current study is available in GEO with the accession number GSE64104, www.ncbi.nlm.nih.gov/geo/query/acc.cgi?acc=GSE64104.

**Electronic supplementary material:**

The online version of this article (doi:10.1007/s00125-017-4261-z) contains peer-reviewed but unedited supplementary material, which is available to authorised users.

## Introduction

Adipose tissue in obese individuals is characterised by adipocyte hypertrophy, oxidative stress, macrophage infiltration and enhanced production of proinflammatory cytokines. The proinflammatory state of adipose tissue significantly contributes to the development of insulin resistance by interfering with insulin signalling routes [1–3]. However, the mechanisms by which local metabolic disturbances in adipose tissue in obesity lead to macrophage infiltration and the onset of chronic inflammation are not fully understood. Recently, the mitochondrial metabolite succinate has been suggested as a potential link between metabolic stress and activation of inflammatory signals [4]. Under non-stressed conditions, succinate is formed from succinyl-CoA by succinyl-CoA synthetase, and is converted into fumarate within the tricarboxylic acid (TCA) cycle. Under conditions of oxidative stress, alterations in functioning of the TCA cycle lead to mitochondrial release of succinate into the cytosol and subsequently the extracellular environment [5–7]. Circulating succinate levels are elevated in rodent models of metabolic disease [8]. Importantly, with the identification of G-coupled receptor 91 (GPR91), also known as succinate receptor 1 (SUCNR1), which is present on the plasma membrane of various cell types, it became clear that succinate may have an important signalling function, providing feedback between local tissue metabolism, mitochondrial stress and organ function [9, 10]. High levels of SUCNR1 are reported in metabolically active tissues such as kidney, adipose tissue and liver [11]. However, the function of the receptor in these tissues, especially under conditions of metabolic stress, remains largely unknown.

Interestingly, SUCNR1 is also expressed in immune cells [11, 12] and stimulation of immune cells with lipopolysaccharide (LPS) does not only activate inflammatory processes, such as secretion of cytokines (e.g. IL-1β), but also coincides with intracellular accumulation [4] and cellular release of succinate [13]. We aimed to evaluate whether SUCNR1 and its ligand, succinate, have an important role in the development and progression of obesity-induced inflammation and insulin resistance in type 2 diabetes. We set out to investigate the role of the SUCNR1 pathway using both animal and human studies combined with various in vitro approaches.

## Methods

### Human studies

Human subcutaneous adipose tissue and visceral adipose tissue samples were collected and digested using collagenase type 1 (Gibco, Thermo Fisher Scientific, Waltham, MA, USA) to isolate mature adipocytes (MA) and the stromal vascular fraction (SVF) as described [14]. The cellular fractions were subsequently used for RNA isolation and reverse transcriptase quantitative PCR (RT-qPCR) analysis. Increased gene expression of *Fabp4* and *Emr1* in the adipocytes and SVF, respectively, confirmed the purity of the fractions (data not shown). Plasma succinate levels were determined in individuals with type 2 diabetes (*n* = 45) and non-diabetic control participants (*n* = 72). Participants were 40–70 years old and those with type 2 diabetes were treated according to national guidelines. All participants with type 2 diabetes had poor glycaemic control while on oral glucose-lowering agents [15].

Blood was taken after an overnight fast in all individuals, just before the start of insulin treatment in the participants with type 2 diabetes. See electronic supplementary material (ESM) for further details and ESM Table 1 for the characteristics of both groups. All participants gave written informed consent and the studies were approved by the ethical committee of the Radboud University Medical Center, Nijmegen, the Netherlands.

### Animal studies

*Sucnr1* ^+/−^ mice on a C57BL/6 background were a kind gift from Amgen (Thousand Oaks, CA, USA) and were generated as described previously [9]. To generate the *Sucnr1* ^+/−^ mice, an IRES/*lacZ*/neo cassette was exchanged for a large part of exon 2, encoding most of the receptor, by homologous recombination. See ESM Fig. 1 and ESM Methods for further details. The animals were intercrossed to yield homozygous *Sucnr1* ^*−*/−^ wild-type (WT) littermate offspring. Mice were housed under standardised conditions (12 h dark/12 h light cycle). Unless stated otherwise, mice were fed ad libitum. Animal experiments were approved by the Animal Experiments Committee of the Radboud University Medical Center.

Eight to 12-week-old male mice were randomised according to body weight and divided into four groups: WT or *Sucnr1* ^*−*/−^ mice receiving either a low-fat diet (LFD; 10% energy derived from fat; D12450J, Research Diets, New Brunswick, NJ, USA) or a high-fat diet (HFD; 60% energy derived from fat; Research Diets, D12492). Each of the four groups was further randomised into three groups that received either 2, 8 or 16 weeks of diet. The investigator was blinded to group assignment and outcome assessment. Body weight was measured weekly and GTTs were performed at the end of weeks 2, 8 or 16 as described below. Finally, the mice were anaesthetised with isoflurane for collection of blood in heparin tubes (BD, Franklin Lakes, NJ, USA) by orbital extraction. After the animals were euthanised by cervical dislocation, epididymal white adipose tissue and liver were isolated for further analysis. Tissues were fixed for immunohistochemistry using 4% (vol./vol.) formaldehyde and embedded in paraffin. The remaining tissue was snap frozen in liquid nitrogen for RNA extraction.

### Glucose tolerance test

After 11 h of fasting, mice were subjected to a GTT, receiving an intraperitoneal injection of glucose solution (2 g glucose/kg body weight). After 15, 30, 60 and 120 min, blood was collected from the tail and glucose was measured using Accu-Chek glucose readers (Roche, Mannheim, Germany). The investigator was blinded to the genotype.

### Isolation and culture of white adipose tissue explants

Mouse epididymal adipose tissue from 12-week-old C57BL/6 mice was freshly isolated and 0.2 g tissue was directly brought into culture in 1 ml isotonic 10 mmol/l HEPES buffer. Samples were incubated for 16 h under normoxic (20% O_2_) or hypoxic (1% O_2_) conditions in the presence of various glucose concentrations. See ESM for culture of obese vs lean white adipose tissue explants.

### Macrophage and adipose tissue co-culture

Bone marrow-derived macrophages (BMDMs) were obtained from C57Bl/6 mice, differentiated and exposed to a transwell chamber (0.4 μm, Corning, Corning, NY, USA) containing adipose tissue explants. See ESM for further details.

### Morphologic analysis of adipose tissue and quantification of macrophage number

H&E staining of sections followed standard protocols. Morphometric analysis of individual fat cells was done using digital image analysis software, as described previously [16]. To quantify macrophage numbers, sections were immunohistochemically stained for F4/80, as described previously [14]. See ESM for further details.

### Succinate measurements

Succinate concentration in plasma was measured with a modified protocol for the Succinic Acid Kit (Megazyme, Bray, Ireland). See ESM for further details.

### ^1^H NMR spectroscopy

One-dimensional ^1^H NMR spectroscopy was performed to investigate the concentration of succinate in the medium from the samples. See ESM for further details.

### Plasma metabolites

Plasma metabolites were measured in a fasted state. Cholesterol, triacylglycerols, glucose (Liquicolor, Human, Wiesbaden, Germany) and NEFA (NEFA-C, Wako Chemicals, Neuss, Germany) were measured enzymatically. Plasma insulin (ultrasensitive mouse insulin ELISA kit, Crystal Chem, Downers Grove, IL, USA) and leptin (R&D Systems, Minneapolis, MN, USA) were measured by ELISA according to the manufacturer’s instructions.

### Liver lipids

Hepatic triacylglycerol concentrations were measured in 10% (wt./vol.) liver homogenates using a commercial kit from Liquicolor (Human) and expressed per mg protein as determined by bicinchoninic acid (BCA) protein assay (Thermo Fisher Scientific, Rockford, IL, USA). For histological examination, H&E staining of liver sections, 5 μm thickness, followed standard protocols.

### In vitro cytokine production

Cytokine secretion was determined in peritoneal cells and BMDMs from *Sucnr1* ^*−*/*−*^ and WT mice. See ESM for further details.

### Transwell chemotaxis assay

BMDMs were obtained from 3–4-month-old mice and differentiated for 7 days in DMEM with 10% (vol./vol.) serum, supplemented with 30% (vol./vol.) L929-conditioned medium. BMDM migration assays were performed using 8.0 μm pore size 24 well Transwell chambers (BD Biosciences, Bedford, MA, USA); see ESM for further details.

### RNA isolation and RT-qPCR analysis

RNA isolation and real-time RT-qPCR was used to determine the relative expression levels of mRNAs. See ESM for further details and ESM Table 2 for primer sequences.

### Microarray analysis and biological interpretation of array data

Epididymal adipose tissue samples from LFD WT and *Sucnr1* ^−/−^ animals (*n* = 4 per genotype) were subjected to genome-wide expression profiling using Affymetrix Mouse Gene 1.1 ST arrays (Affymetrix, Santa Clara, CA, USA). Details on RNA isolation, integrity controls, hybridisation and statistical analysis, as well as biological interpretation of array data, can be found in the ESM. Array data have been submitted to the Gene Expression Omnibus under accession number GSE64104. Expression patterns of SUCNR1 in murine and human macrophages were extracted from publicly available microarray datasets (GSE69607 and GSE5099, respectively).

### Statistical analysis

All values are expressed as mean ± SEM. Data were only excluded in the case of technical failure assessed by positive and negative control values. Statistical comparisons between two groups were calculated using a Student’s *t* test. Differences between four groups were tested with ANOVA, followed by post hoc Bonferroni correction. A value of *p* < 0.05 was regarded as statistically significant.

## Results

### Hypoxia and hyperglycaemia induce succinate release from adipose tissue

**Fig. 1:**
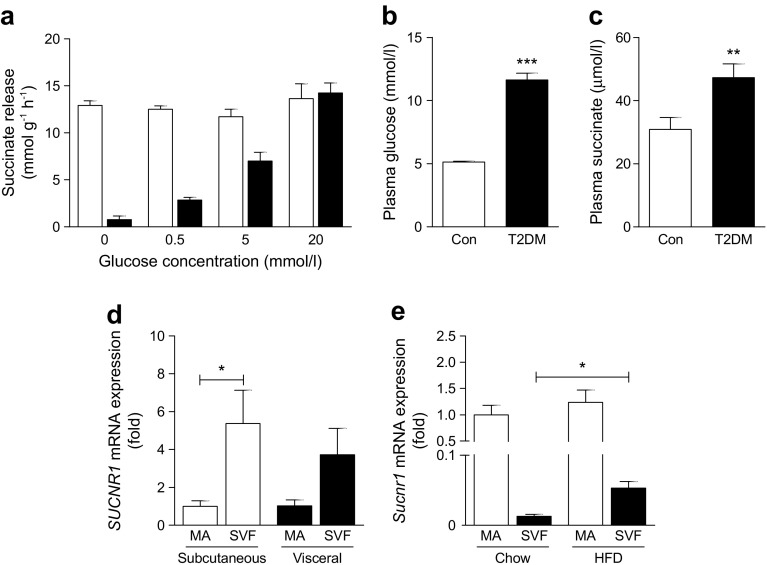
Succinate is released from adipose tissue during hypoxia and hyperglycaemia. (**a**) Succinate release from mouse adipose tissue explants incubated under hypoxic (white bars) or normoxic (black bars) conditions in medium with increasing glucose concentration. (**b**) Plasma glucose levels and (**c**) plasma succinate levels in control participants (*n* = 76) and type 2 diabetes patients (*n* = 58). (**d**) *SUCNR1* mRNA levels in MA and SVFs of human subcutaneous and visceral adipose tissue (*n* = 7). Data are fold change compared with MA subcutaneous. (**e**) *Sucnr1* mRNA levels in MA and SVF of epididymal adipose tissue from mice fed chow or HFD (*n* = 4). Data are fold change compared with MA from chow-fed mice. Con, control; T2DM, type 2 diabetes. ^*^ *p* < 0.05, ^**^ *p* < 0.01 and ^***^ *p* < 0.001 vs control or as indicated

Obese insulin-resistant adipose tissue is characterised by a hyperglycaemic and hypoxic environment [17, 18]. To evaluate whether these conditions influence succinate secretion, adipose tissue explants were incubated in medium with increasing glucose concentrations under both normoxic and hypoxic conditions (1% oxygen tension). Under normoxic conditions, increasing glucose concentrations induced a dose-dependent increase in succinate release (up to 18-fold) (Fig. 1a). Hypoxia markedly increased succinate release from adipose tissue (17-fold) compared with normoxia, but independently of glucose concentration (Fig. 1a). No significant differences were found in succinate release between lean and obese adipose tissue ex vivo (ESM Fig. 2a). In line with this, participants with diabetes and hyperglycaemia (Fig. 1b) had a 53% increase in circulating succinate level compared with normoglycaemic individuals (Fig. 1c). No significant correlations were found, however, between plasma succinate levels and plasma glucose levels or other patient characteristics (ESM Table 3).

We subsequently set out to evaluate the significance of SUCNR1 in adipose tissue. Human adipose tissue had higher *SUCNR1* mRNA expression in the SVF compared with MA (Fig. 1d). In contrast, murine adipose tissue revealed higher *Sucnr1* mRNA in MA vs the SVF (Fig. 1e). Interestingly, however, feeding mice an HFD rather than regular chow increased expression of *Sucnr1* in the SVF (Fig. 1e), pointing towards a role of SUCNR1 in immune cell function in enlarged adipose tissue. Obese adipose tissue is characterised by an increased M1/M2 macrophage ratio [19]. To evaluate the expression of SUCNR1 in M1 and M2 macrophages, publicly available datasets of murine (GSE69607) [20] and human (GSE 5099) [21] macrophages were analysed. Murine *Sucnr1* expression is similar in bone-marrow-derived (LPS-stimulated) M1 and (IL-4 stimulated) M2 macrophages compared with (medium control) M0 macrophages (ESM Fig. 2b). In human cells, *SUCNR1* expression was increased in M2 compared with M1 macrophages (ESM Fig. 2c) and was enhanced after differentiation from monocytes to macrophages (ESM Fig. 2d). This reveals differences in SUCNR1 expression patterns between mouse and human macrophages. Interestingly, co-culture of murine BMDMs with adipose tissue explants increased expression of *Sucnr1* in the macrophages (ESM Fig. 2e), showing that the presence of adipose tissue rather than the macrophage M1/M2 phenotype determines SUCNR1 expression. There was no difference in *Sucnr1* expression between macrophages exposed to explants from lean vs obese mice (ESM Fig. 2e).

### Absence of SUCNR1 affects inflammatory processes in adipose tissue

**Fig. 2:**
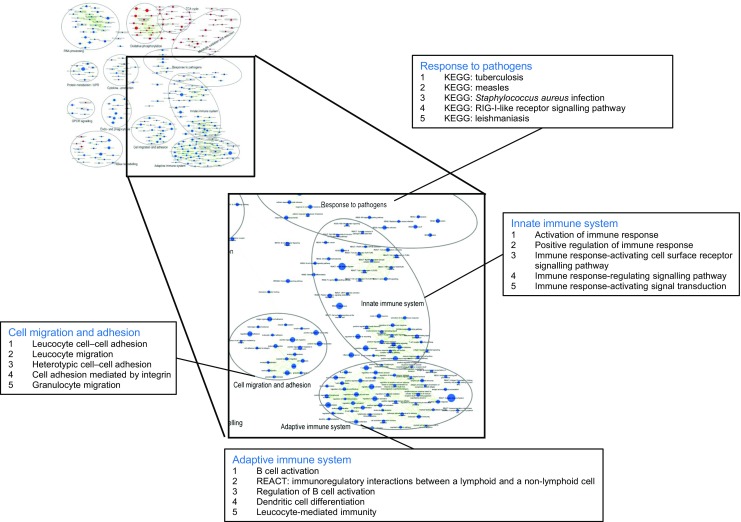
Absence of SUCNR1 reduces inflammatory pathways within adipose tissue. Enrichment map zooming in on four differentially regulated pathways in adipose tissue from *Sucnr1* ^−/−^ vs WT mice. A larger version of the enrichment map is shown in ESM Fig. 3. Gene set enrichment analysis was performed to identify functional gene sets (i.e. metabolic pathways or signalling transduction routes) that were changed in *Sucnr1* ^−/−^ mice (*p* < 0.001, false discovery rate [FDR] < 0.25). Nodes represent gene sets. A red node indicates induction of a gene set in *Sucnr1* ^−/−^ compared with WT, whereas a blue node indicates suppression of a gene set. Node size represents the gene set size. Gene sets were grouped by cluster analysis, applying the Markov cluster algorithm. For four specific clusters related to inflammation, the five most significant gene sets are shown. KEGG, Kyoto Encyclopedia of Genes and Genomes (www.genome.jp/kegg/); REACT, Reactome (www.reactome.org/); RIG-I, retinoic acid-inducible gene I

Succinate appears to activate inflammatory pathways at least partly via SUCNR1 [4, 22]. Interestingly, microarray data of adipose tissue of LFD-fed *Sucnr1* ^*−*/*−*^ and WT mice revealed that absence of SUCNR1 reduces the inflammatory trait of the adipose tissue (ESM Fig. 3). Pathways assigned to the innate immune system, cell migration and pathogen response are downregulated in adipose tissue of *Sucnr1* ^*−*/*−*^ mice (Fig. 2). These data suggest that the succinate signalling pathway may affect inflammatory processes in adipose tissue.

### Absence of SUCNR1 does not affect body weight gain on HFD feeding in mice, but reduces adipose tissue macrophage infiltration and improves glucose tolerance

**Fig. 3:**
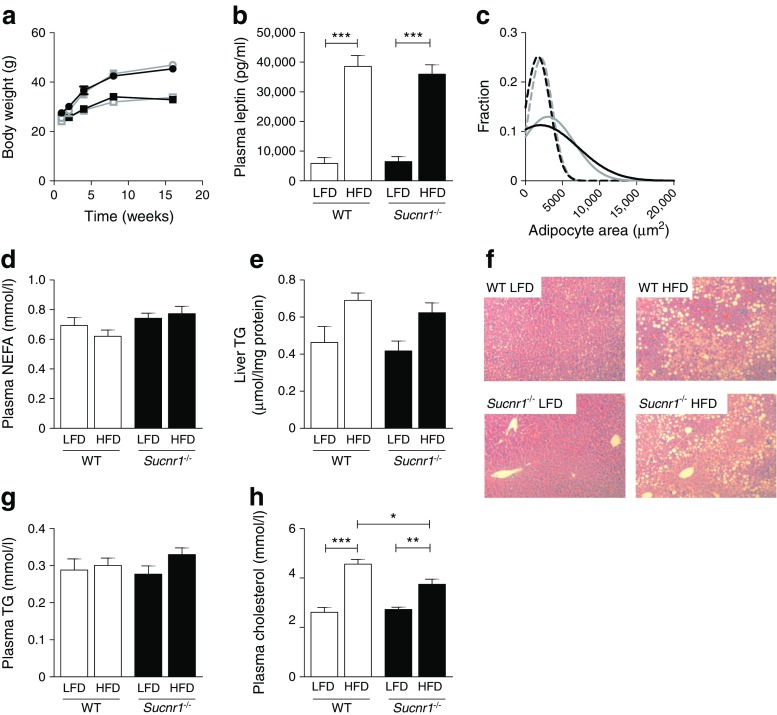
Absence of SUCNR1 does not affect body weight gain on HFD feeding. *Sucnr1* ^−/−^ and WT mice were subjected to LFD or HFD feeding for 16 weeks. (**a**) Body weight development over time. Grey, WT; black, *Sucnr1* ^−/−^; squares, LFD; circles, HFD. (**b**–**h**) Results after 16 weeks of LFD or HFD feeding: (**b**) plasma leptin levels; (**c**) adipocyte size distribution of epididymal adipose tissue; the best Gaussian fit curve is shown for the level of adipocytes (fraction) within each 1000 μm^2^ area segment (grey, WT; black, *Sucnr1* ^−/−^; dashed line, LFD; solid line, HFD); (**d**) plasma NEFA; (**e**) hepatic triacylglycerol content; (**f**) H&E staining of liver sections (magnification ×200); (**g**) plasma triacylglycerol; and (**h**) plasma cholesterol. Data are mean ± SEM from *n* = 7 animals per group. ^*^ *p* < 0.05, ^**^ *p* < 0.01 and ^***^ *p* < 0.001. TG, triacylglycerol

To determine whether succinate signalling via SUCNR1 contributes to obesity-induced abnormalities in adipose tissue, *Sucnr1* ^*−*/*−*^ and WT littermates were subjected to HFD to induce obesity, or to LFD as a control diet. Noticeably, during the development of obesity in WT mice, *Sucnr1* expression levels in adipose tissue did not change (data not shown). WT and *Sucnr1* ^*−*/*−*^ animals had similar body weights after 16 weeks of HFD (Fig. 3a), and an equal increase in plasma leptin levels (Fig. 3b). In addition, no differences in epididymal adipose tissue mass (ESM Fig. 4a) or adipocyte size (Fig. 3c and ESM Fig. 4b) were observed between the genotypes, paralleled by similar plasma NEFA levels (Fig. 3d).

**Fig. 4:**
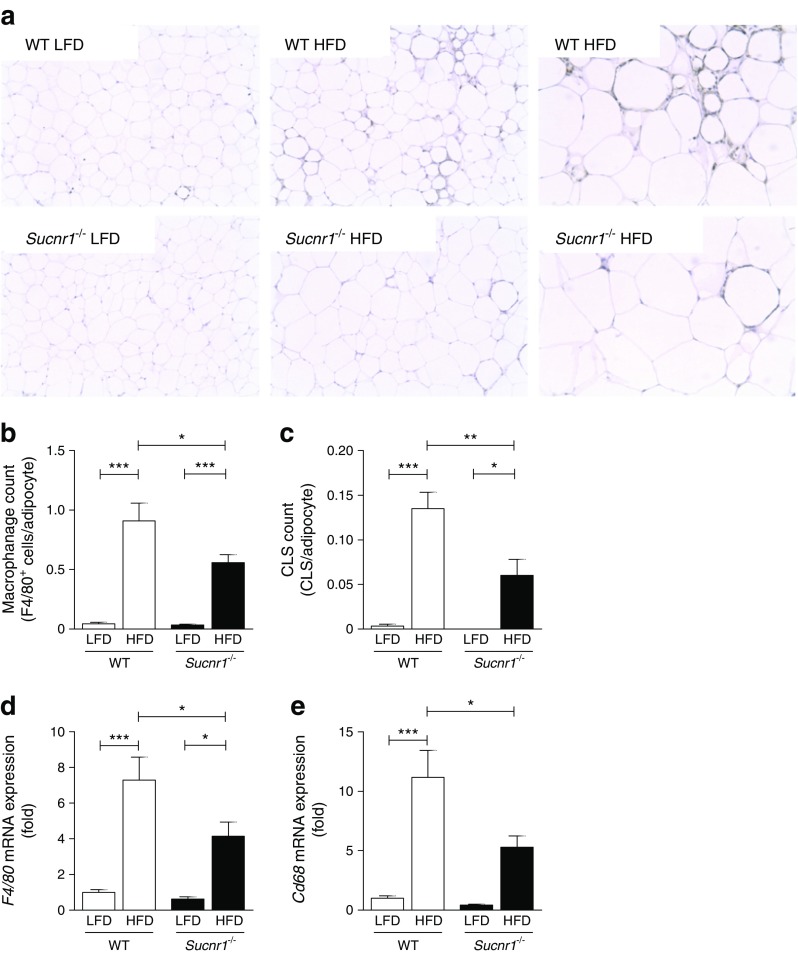
In mice, absence of SUCNR1 reduces infiltration of macrophages into white adipose tissue in obesity. (**a**) Macrophage influx into adipose tissue as determined by immunohistochemistry, with F4/80 staining (×100 and ×200 magnification). Quantification of (**b**) macrophages and (**c**) mean CLS, expressed as number per adipocyte. Relative mRNA levels of (**d**) *F4/80* and (**e**) *Cd68* in epididymal white adipose tissue of *Sucnr1* ^−/−^ and WT mice after LFD or HFD feeding for 16 weeks. mRNA levels are expressed as fold change compared with WT mice fed an LFD. Data are mean ± SEM from *n* = 6–7 animals per group. ^*^ *p* < 0.05, ^**^ *p* < 0.01 and ^***^ *p* < 0.001

The livers of HFD WT and *Sucnr1* ^*−*/*−*^ animals showed similar levels of steatosis, reflected by hepatic triacylglycerol levels (Fig. 3e) and H&E staining (Fig. 3f). Plasma triacylglycerol levels were similar in WT and *Sucnr1* ^*−*/*−*^ animals (Fig. 3g). Total plasma cholesterol levels increased in response to HFD feeding, but the increase was less in HFD-fed *Sucnr1* ^*−*/*−*^ vs WT animals (Fig. 3h). Analysis of inflammatory pathways in adipose tissue revealed a lower number of F4/80^+^ macrophages in adipose tissue from HFD-fed *Sucnr1* ^*−*/*−*^ mice compared with HFD-fed WT animals (Fig. 4a, b), paralleled by a reduced number of crown-like structures (CLS) (Fig. 4c). RT-qPCR analysis for the macrophage markers *F4/80* (also known as *Adgre1*) (Fig. 4d) and *Cd68* (Fig. 4e) confirmed these results. This statistically significant reduction in macrophage numbers was apparent after 16 weeks of an HFD, but not by 8 weeks (ESM Fig. 5a, b).

**Fig. 5:**
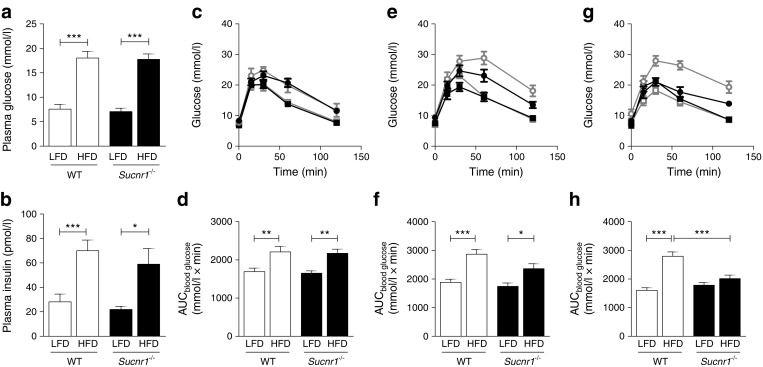
Absence of SUCNR1 improves glucose tolerance on HFD feeding. *Sucnr1* ^−/−^ and WT mice were subjected to LFD or HFD feeding for 16 weeks (*n* = 7). (**a**) Plasma glucose and (**b**) insulin levels, 4 h fasted after 16 weeks of HFD feeding. Blood glucose levels and AUC during GTTs, overnight fasted after 2 weeks (**c**, **d**), 8 weeks (**e**, **f**) and 16 weeks (**g**, **h**) of LFD or HFD feeding. White/grey, WT; black, *Sucnr1* ^−/−^; squares, LFD; circles, HFD. ^*^ *p* < 0.05, ^**^ *p* < 0.01 and ^***^ *p* < 0.001

We assayed glucose metabolism after 2, 8 and 16 weeks of HFD feeding (Fig. 5). Plasma glucose and insulin levels were increased after 16 weeks of HFD compared with LFD feeding, but did not differ between genotypes (Fig. 5a, b).

Glucose tolerance was similar in WT and *Sucnr1* ^*−*/*−*^ mice after 2 weeks of HFD feeding (Fig. 5c, d). After 8 weeks of HFD feeding, glucose intolerance developed in HFD-fed WT animals, while *Sucnr1* ^−/−^ mice tended to remain glucose tolerant (Fig. 5e, f). This difference was more pronounced and highly significant after 16 weeks of HFD feeding (Fig. 5g, h). We conclude that the absence of SUCNR1 protects against the development of obesity-induced adipose tissue inflammation and glucose intolerance.

### Absence of SUCNR1 does not affect macrophage cytokine production, but reduces chemotaxis towards apoptotic and hypoxic adipocytes

To determine whether SUCNR1 has any direct effect on the production of cytokines, peritoneal macrophages from WT and *Sucnr1* ^*−*/*−*^ animals were stimulated with inflammatory LPS. Absence of SUCNR1 did not affect intracellular IL-1β levels or secretion of TNF-α or IL-6 (ESM Fig. 6a–c). In BMDMs, succinate itself did not affect secretion of the chemokine (C-X-C motif) ligand 1 (KC or CXCL1), either in the absence or presence of LPS (ESM Fig. 6d). Moreover, absence of SUCNR1 did not alter secretion of KC in response to LPS or succinate, suggesting no direct role of this pathway in the cytokine or chemokine secretion potential of macrophages. In accordance, absence of SUCNR1 did not affect the expression of the chemokine *Mcp1*, nor the (anti)-inflammatory phenotype of macrophages in adipose tissue after 16 weeks of HFD feeding, illustrated by unchanged expression of *Casp1*, *TNFα* (also known as *Tnf*), *Cd86*, *Cd80*, *iNos* (also known as *Nos2*), *Mrc1* and *IL-1ra* (also known as *Il1rn*) (ESM Fig. 7a–h).

**Fig. 6:**
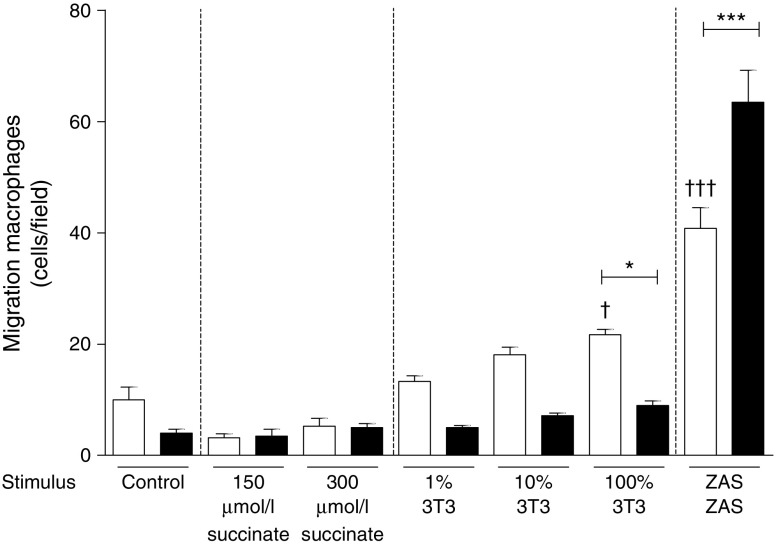
Absence of SUCNR1 improves glucose tolerance on HFD feeding. Migration of BMDMs from *Sucnr1* ^−/−^ (black bars) and WT (white bars) mice towards succinate, medium derived from apoptotic and hypoxic 3T3-L1 adipocytes (3T3) and ZAS. Data are mean ± SEM; *n* = 4–6. ^*^ *p* < 0.05 and ^***^ *p* < 0.001 compared with WT. ^†^ *p* < 0.05 and ^†††^ *p* < 0.001 compared with WT control

The absence of SUCNR1 reduced the number of macrophages in obese adipose tissue. Hypoxia/hyperglycaemia induced the release of succinate by adipose tissue. As succinate may serve as a chemoattractant, we investigated its role and that of its receptor in the chemotactic response of macrophages (Fig. 6). Macrophages of both genotypes showed highly induced migration towards the positive control (zymosan-activated serum [ZAS]), with *Sucnr1* ^−/−^ macrophages showing the highest number of migrated macrophages/field. Of note, no migration towards succinate alone was observed. In contrast, using medium derived from apoptotic and hypoxic 3T3 adipocytes as chemotactic factor, macrophages lacking SUCNR1 showed reduced migration potential. The succinate concentration in medium from apoptotic and hypoxic 3T3 adipocytes was 14% and 33% higher, respectively, than in medium from control cells (ESM Fig. 8).

We evaluated whether the absence of SUCNR1 affects expression of receptors involved in chemotaxis in BMDMs and adipose tissue derived from *Sucnr1* ^−/−^ and WT mice (ESM Fig. 9). We did not detect major differences in the expression of *Ccr5*, *Cxcr4* or *Cd74* in BMDMs, but *Ccr1* expression was reduced in *Sucnr1* ^−/−^ cells. In contrast, in adipose tissue, expression of *Ccr1* was not different, while *Cxcr4* and *Cd74* were upregulated on HFD feeding in WT mice, but not in *Sucnr1* ^−/−^ mice; *Ccr5* expression was increased in *Sucnr1* ^−/−^ compared with WT controls fed an LFD.

## Discussion

Our results demonstrate that succinate release from adipose tissue is induced under metabolically disturbed conditions, including hyperglycaemia and hypoxia. Moreover, we find that the succinate signalling pathway is involved in the migration of macrophages towards adipose tissue, a crucial step in the development of obesity-induced adipose tissue inflammation leading to systemic glucose intolerance.

### Increased succinate release by adipose tissue in response to hypoxia and hyperglycaemia

Excessive release of succinate from adipose tissue was induced by hypoxia or hyperglycaemia. Surprisingly, ex vivo release of succinate from obese adipose tissue was not greater than that from lean adipose tissue. Possibly, succinate release from adipose tissue is acutely induced by stress factors such as hypoxia and hyperglycaemia, which may similarly occur (locally) in obese adipose tissue in vivo. However, we envisage that ex vivo culturing of obese and lean adipose tissue in stable circumstances diminishes differences that may occur in vivo.

Circulating succinate levels were elevated in patients with type 2 diabetes, suggesting that plasma succinate levels may be sustained in chronic stress conditions such as obesity. Our findings are confirmed by recent findings of elevated succinate levels in plasma and adipose tissue from obese diabetic mouse models [8] [23]. In contrast to our data, a previous study did not find any increase in serum succinate in individuals with type 2 diabetes [8]. This discrepancy could possibly be explained by differences in individual characteristics. Participants with type 2 diabetes in our study were selected based on failure of glycaemic control, and had relatively high fasting plasma glucose levels. However, there was no significant correlation of plasma succinate with fasting plasma glucose levels, BMI or plasma lipids. Possibly, our human cohort lacks sufficient power to link plasma glucose levels with succinate levels directly because of the relatively high variation in both variables. Alternatively, our ex vivo data suggest that adipose tissue contributes to the release of succinate under hyperglycaemic conditions. In plasma, however, succinate occurs as the result of secretion by other metabolically active organs, such as liver and kidney, which may override release of succinate by adipose.

Additionally, the intestinal microbiomal *Bacteroides* spp., abundantly present in obese animals, are major producers of succinate and may contribute to circulating succinate levels [24]. Besides metabolic sources, the increased level of inflammation in obesity may further contribute, as inflammatory stimuli have been shown to enhance intracellular succinate levels within macrophages in vitro [4].

### SUCNR1 activation mediates adipose tissue macrophage infiltration and glucose intolerance in obesity

SUCNR1 only needs a twofold increase in plasma or urinary succinate concentrations for a half-maximal response [6, 9]. The effects of SUCNR1 signalling in adipose tissue were largely unknown, yet our results suggest a prominent role for SUCNR1 in the migration of macrophages towards obese adipose tissue. High levels of SUCNR1 expression by dendritic cells and macrophages have been reported previously [22] and likely contribute to the SUCNR1 expression observed within the SVF of adipose tissue. In mice, SUCNR1 expression within the SVF appeared low compared with adipocytes. SUCNR1 may nevertheless have an important signalling function, especially during obesity-induced macrophage infiltration in adipose tissue; this is supported by our in vitro data showing enhanced SUCNR1 expression in murine macrophages exposed to adipose tissue explants. Remarkably, explants from obese mice did not increase SUCNR1 expression more than explants from lean mice, while HFD feeding enhanced SUCNR1 expression within the SVF of adipose tissue. Apparently, factors secreted by adipose tissue induce expression of *Sucnr1* in macrophages in vitro, while in vivo other factors in adipose tissue in HFD mice further enhance expression of *Sucnr1*. Future research should evaluate whether SUCNR1 expression in the SVF is similarly increased in people with type 2 diabetes compared with controls, especially as our data revealed higher *SUCNR1* mRNA levels in the SVF vs adipocytes in humans.

Succinate has previously been identified as a chemoattractant for dendritic cells, with signalling via the SUCNR1 [22]. Despite substantial SUCNR1 expression on macrophages [22], we were unable to show any chemoattractant potential for succinate alone. In line with this, despite the migration observed with medium from apoptotic/hypoxic cells, the succinate concentration in medium from 3T3 cells was low and the difference in concentration was greater between medium from healthy 3T3 cells and control medium than between medium from stressed vs healthy cells. Importantly, the migration of U937 cells towards succinate previously published [22] was very modest (8% of input cells) at a nearly saturating concentration of succinate (150 μmol/l). We and others found that the E_max_ (concentration of succinate at which all SUCNR1 receptors are bound and activated) is around 200 μmol/l. For dendritic cells, only 10% and 35% of input cells responded to E_max_ (225 μmol/l) and supra E_max_ (450 μmol/l) succinate concentrations, respectively. Moreover, another study showed that around E_50_ succinate concentrations (corresponding to 50% E_max_; ~100 μmol/l) did not affect migration of peritoneal macrophages [25], in line with our observations in BMDMs. Thus, although a macrophage cell line has previously shown migration towards succinate, we and others could not confirm this using primary macrophages. This suggests that other factors secreted by apoptotic/hypoxic adipocytes, known to be present in enlarged adipose tissue in obesity [26, 27], induce macrophage migration and that release of succinate from macrophages and auto/paracrine activation of macrophage SUCNR1 synergises with the initial factor to stimulate macrophage migration towards lipid-rich particles. A similar mechanism has been proposed for microglial cell accumulation in age-related macular degeneration, as SUCNR1-deficient microglial cells show impaired migration towards oxidised LDL (and are unresponsive to succinate) [25]. Alternatively, SUCNR1 activation by succinate may induce cytoskeletal changes and polarisation of immune cells facilitating their migration.

Although *Sucnr1* ^*−*/−^ BMDMs showed reduced expression of chemokine (C-C motif) receptor 1 (CCR1), we propose that the reduced migration of *Sucnr1* ^*−*/−^ BMDMs is most likely not caused by reduced expression of CCR1 alone. Notably, migration per se towards ZAS as chemoattractant was not altered in BMDMs lacking SUCNR1. However, specific migration to conditioned adipocyte medium was affected in the absence of SUCNR1.

SUCNR1 deficiency does not affect pro- and anti-inflammatory markers in adipose tissue derived from HFD-fed mice or cytokine production in response to succinate and/or LPS in vitro. In dendritic cells, previous studies showed that succinate acts in synergy with toll-like receptor (TLR) ligands to potentiate the production of proinflammatory cytokines, at least partly via SUCNR1 [22]. Our results suggest that succinate does not have similar effects on (adipose tissue) macrophages. The improvement of the adipose tissue inflammatory trait in the absence of the SUCNR1, as evidenced by our microarray analysis, can therefore most likely be explained by other mechanisms, including a reduction in the absolute number of macrophages.

We show that *Sucnr1* ^*−*/−^ mice display enhanced glucose tolerance on HFD feeding, despite having body weight similar to WT mice. This enables the study of the role of SUCNR1 in obesity-induced inflammation and metabolic disturbances independent of changes in body weight or adipose tissue weight. A recent study showed that *Sucnr1* ^*−*/−^ mice have increased body weight, with concurrent hyperglycaemia and impaired glucose tolerance [28], in contrast to our observations. Strikingly, the results of this group varied with the type and length of HFD feeding. Only a prolonged dietary HFD intervention of 20 weeks caused metabolic disturbances, while chow-fed *Sucnr1* ^*−*/−^ mice had reduced adipose tissue weight. Moreover, even HFD feeding for only 11 weeks reduced body weight and tended to improve glucose tolerance [28]. The dichotomous effects of SUCNR1 on the development of obesity in their experiments could possibly result from the specific Cre model used to generate their *Sucnr1* ^*−*/−^ mouse or the different diets/intervention periods [28]. The authors suggest that the glucose intolerance likely concurs with the increased white adipose tissue weight after prolonged HFD feeding. The improvement in glucose tolerance in *Sucnr1* ^*−*/−^ mice can be the result of improved insulin sensitivity and/or reduced insulin secretion, something we cannot distinguish using our data. In addition, as we used total *Sucnr1* ^*−*/−^ mice, we cannot distinguish the effects of SUCNR1 deficiency in individual cell types, such as adipocytes vs macrophages. Future studies are needed to disentangle these effects.

Overall, our results have identified succinate and its receptor as a driver of obesity-induced inflammation and an important contributor to the migration of macrophages into adipose tissue, leading to the systemic glucose intolerance in obesity-induced type 2 diabetes. As such, our data put forward SUCNR1 as a promising therapeutic target to combat obesity-induced diabetes.

## Electronic supplementary material

ESM (PDF 1886 kb)

## Abbreviations

BMDMs: Bone marrow-derived macrophages
CLS: Crown-like structures
HFD: High-fat diet
LFD: Low-fat diet
LPS: Lipopolysaccharide
MA: Mature adipocytes
SUCNR1: Succinate receptor 1
SVF: Stromal vascular fraction
TCA: Tricarboxylic acid
WT: Wild-type
ZAS: Zymosan-activated serum
