# Supplementary material for: SUCNR1-mediated chemotaxis of macrophages aggravates obesity-induced inflammation and diabetes
Source: Diabetologia. 2017 Apr 5;60(7):1304–13. doi: 10.1007/s00125-017-4261-z (PMC5487589; doi:10.1007/s00125-017-4261-z)
Supplement: Supplementary file 1 — (PDF 1886 kb) [file 125_2017_4261_MOESM1_ESM.pdf]

ESM METHODS

Human studies – type 2 diabetes patients and controls

Subjects were between 40 and 70 years old and individuals with type 2 diabetes were treated according to national guidelines. All participants with type 2 diabetes had poor glycaemic control while on oral glucose-lowering agents and diet (79% of the individuals with type 2 diabetes wereusing Metformin; 69% using Sulfonylureas). Blood was taken after an overnight fast, just before the start of insulin treatment in the participants with type 2 diabetes. Control participants were recruited through advertisements in local newspapers. Subjects characteristics are listed in Supplementary Table S1. Exclusion criteria were other types of diabetes, significant cardiovascular, renal, liver or other co-morbidity, use of corticosteroids, uncontrolled endocrine disorders (stable supplementation with thyroid hormone was allowed), bariatric treatment, excessive alcohol consumption (>20 g/day), drug abuse, and use of thiazolidinedione derivatives.

Sucnr1$^{-/-}$ **mice**

To generate the *Sucnr1*$^{+/-}$ mice, an IRES/lacZ/neo cassette was inserted to delete a large part of exon 2, by homologous recombination. See ESM Fig. 1 for further details. The animals were intercrossed to yield homozygous wild-type (WT) and *Sucnr1*$^{-/-}$ offspring as determined by *Sucnr1* genotyping using the primers TL23-F1: GCTGTCTGGGCCTTAGTGACC, TL-23-R1: GCTGCCTTCTGATTCATGTGG, Neo3a: GCAGCGCATCGCCTTCTATC.

Isolation and culture of obese vs lean white adipose tissue explants

Mouse epididymal adipose tissue was freshly isolated from obese mice as well as age-matched lean control mice. Obese mice received high fat diet (HFD) containing 45% energy derived from fat (58V8 Test Diet, St Louis, USA) + 1% cholesterol (Dishman, Veenendaal, Netherlands) and 20% fructose (wt/vol) in the drinking water for 18 weeks. Lean mice were

age-matched chow fed mice. 0.2 g of tissue was directly brought into culture in 1 ml DMEM containing 1 mmol/l glucose for 24 hours.

Macrophage and adipose tissue co-culture

Bone marrow-derived macrophages (BMDMs) were obtained from C57Bl/6 mice and differentiated for 3 days in DMEM with 10% serum, supplemented with 5% L929 conditioned medium. BMDMs were subsequently plated in 24-wells plates and exposed to a transwell chamber (0.4 µm Corning) containing 50 mg adipose tissue explants (BMDMs with adipose tissue) or an empty transwell chamber (BMDMs without adipose tissue) for another 3 days. BMDMs were subsequently scraped for isolation of RNA and RT-qPCR analysis.

Morphologic analysis of adipose tissue and quantification of macrophage number

Hematoxylin and eosin (H&E) staining of sections followed standard protocols on 5 µm-thick sections of white adipose tissue. Morphometric analysis of individual fat cells was done using digital image analysis software. For this, microscopic images were digitized in 24 bit RGB (specimen level pixel size 1.28 × 1.28 um^2) and recognition of fat cells was performed by applying a region-growing algorithm on manually indicated seed points. To quantify macrophage numbers in epididymal white adipose tissue, sections were immunohistochemically stained using a rat anti-mouse F4/80 antibody (Serotec, Düsseldorf, Germany) followed by a biotinylated rabbit anti-rat antibody and an avidin-biotin-complex (ABC) coupled to peroxidase (Vector Labs, Brunschwig Chemie, Amsterdam, the Netherlands). Visualization of the complex was done using 3,3'-diaminobenzidene for 5 min. With negative controls, primary antibodies were omitted. Macrophages and crown-like structures were counted with a microscope at a magnification of 200x, 10 images per tissue per mouse and expressed per number of adipocytes that were counted in the same image (N=3).

Succinate measurements

Reagent solutions were dissolved according to the kit's protocol. Standard curve and 1:5 diluted plasma samples (100 µl) were measured in duplicate in 96 wells plates. A standard curve was generated with the following molarities of succinate: 0-10-20-40-80-120-160-200 µM. Samples were added to a non-enzyme control plate and an enzymatic assay plate. A reaction mix of 33.6 µl per well (10 µl of solution 1, 2 and 3, 1 µl of solution 4, and 2.6 µl of 20 mmol/l Tris pH 7.4) was prepared and added to the sample. Next, 20 µl of a mixture of 5 µl solution 1, 14 µl mQ water and either 1 µl solution 5 or 1 µl mQ was added per well. Abs340 was read every 5 minutes in a plate reader (Biorad Benchmark Plus) until values stabilized. Data was processed by subtracting the abs340 of the enzyme-treated samples from the abs340 of the negative control plate and succinate concentrations were calculated by applying the Δabs340 value in the standard curve.

1H NMR spectroscopy

One-dimensional 1H NMR spectroscopy was performed to investigate the concentration of succinate in the medium from samples. For this, the medium was filtered through a 10 kDa filter, the volume adjusted to 700 µl with water, and pH adjusted to 2.5 using 3M HCl, after which 20 µl of 20.2 mmol/l sodium 3-trimethylsilyl- 2,2,3,3-tetradeuteropropionate (TSP; Aldrich) in D2O (Catalogue No. 435 767; Aldrich) was added. The samples were then placed in 5-mm NMR tubes and 1H NMR spectra were obtained using a Bruker 500 MHz spectrometer (pulse angle, 90°; delay time, 4 s; number of scans, 256). Water resonance was suppressed by gated irradiation centered on the water frequency. The spectral width in the F1 and F2 domains were 5500 Hz. A total of 2K data points were collected in t2, 256 t1 increments with 32 transient per increment were used. The relaxation delay was set to 2 seconds. Before the Fourier transformation, a sine-bell function was applied in both time domains. During the relaxation delay, the water resonance was presaturated.

The free-induction decays measured for these samples were processed using Topspin software (Bruker, Billerica, Massachusetts, USA). Fourier transformation was applied on the free-induction decay of the samples and the resulting spectra were phase and baseline

corrected. The chemical shifts in the spectra were referenced to the internal standard, TSP. Assignment of peak positions for compound identification was performed by comparing the peak positions in the spectra of the metabolites with the reference spectral database of model compounds at pH, 2.5 using Amix version 3.9.14 (Bruker Bio-spin). Quantification of identified compounds was performed by manual integration of chosen peak(s) for a specific metabolite.

In vitro cytokine production

Peritoneal macrophages were isolated from mice by injecting 5 ml of ice-cold sterile PBS (pH 7.4) into the peritoneal cavity. After centrifugation and washing, cells were resuspended in RPMI 1640 culture medium containing 1 mmol/l pyruvate, 2 mmol/l L-glutamine, and 50 mg/liter gentamicin. Cells were counted using a Z1 Coulter particle counter (Beckman Coulter; Woerden, The Netherlands) and cultured in 96-well round-bottom microtiter plates (Costar, Corning, The Netherlands) at 1×10^5 cells/well in a final volume of 200 µl. After 24 h of incubation with LPS (10 ng/mL) at 37°C and 5% CO_2, the plates were centrifuged at 1,400 × *g* for 8 min, and the supernatants were collected and stored at −80°C until cytokine assays were performed.

Mouse bone marrow-derived macrophages (BMDMs) were differentiated for 7 days in DMEM with 10% serum supplemented with 30% L929 conditioned medium. 2×10^5 cells/well were subsequently stimulated in 96-well flat-bottom microtiter plates with various concentrations of succinate (0-30-300-3000 µmol/l) in the presence or absence of LPS (1 ng/ml).

The concentrations of mouse tumor necrosis factor alpha (TNF-α) and IL-1β were determined by specific radioimmunoassay (RIA). Interleukin-6 (IL-6) and keratinocyt-derived chemokine (KC) were measured using mouse IL-6 and KC ELISA kits (R&D Systems, MN, USA) according to the instructions of the manufacturer.

Transwell chemotaxis assay

(BMDMs were obtained from 3-4 month old mice and differentiated for 7 days in DMEM with 10% (vol/vol) serum, supplemented with 30% (vol/vol) L929 conditioned medium. BMDM migration assays were performed using 8.0 µm pore-size 24-well Transwell chambers (BD Biosciences). BMDMs (2 x 10^5 cells/well) were placed in the upper chamber and medium containing the chemoattractant was added in the lower chamber, all diluted in DMEM supplemented with 0.1% (wt/vol) BSA. Chemoattractants used were 1) various concentrations of succinate 2) various concentrations of medium derived from hypoxic and apoptotic 3T3-L1 adipocytes and 3) 10% zymosan activated serum (ZAS) (wt/vol) as a positive control. For the hypoxic/apoptotic 3T3-L1 cell medium, mouse 3T3-L1 cells were cultured and differentiated towards adipocytes as described [1] and subsequently incubated for 24h at 1% O_2 or exposure to 200 mJ UV radiation to induce hypoxia or apoptosis, respectively. For the chemoattractant solution, supernatant of the hypoxic and apoptotic adipocytes was mixed in a 1:3 vol/vol ratio. To generate the ZAS, 5% zymosan (wt/vol) was incubated in serum for 30 min at 37°C, centrifuged and 10x diluted in DMEM supplemented with 0.1% (wt/vol) BSA. After 8 hours of migration, membranes were fixed with 4% formalin and stained with hematoxylin. Non-migrating cells were removed from the upper surface using a cotton swab. Membranes were mounted on microscope slides and the number of migrated cells on the lower surface was determined in 15-20 representative fields (400x magnification). Four to six separate membranes were analyzed for each condition.

RNA isolation and RT-qPCR analysis.

Total RNA was isolated from adipose tissue using TRIzol (Invitrogen, Carlsbad, CA), according to manufacturer’s instructions. RNA was reverse-transcribed (iScript cDNA Synthesis Kit, Bio-Rad Laboratories). The following qPCR was performed using power SYBR green master mix (Applied Biosystems, Foster City, CA) using the StepOne Real-Time PCR System (Applied Biosystems, Foster City, CA). For mice samples, we used 36B4 and cyclophillin as housekeeping genes to normalize the mRNA quantities. For human samples,

we used beta-2-microglobulin (B2M) as a housekeeping gene. Specific primer sequences used are listed in Supplemantary Table S2.

Microarray analysis.

Epididymal adipose tissue samples from low fat diet (LFD)-fed WT and *Sucnr1*$^{-/-}$ animals (n=4 per genotype) were subjected to genome-wide expression profiling. In brief, total RNA was isolated from adipose tissue samples and integrity was confirmed using a Bio-analyzer (Agilent). Subsequently, RNA was hybridized on Affymetrix Mouse Gene 1.1 ST arrays (Affymetrix, Santa Clara, CA). Packages from the Bioconductor project [2], integrated in an online pipeline [3], were used for quality control and statistical analysis of the array data. Probe sets were first redefined utilizing current genome annotation information [4]. Probes were reorganized based on the gene definitions available in the GRCm38.p2 mouse genome assembly released by the Genome Reference Consortium (remapped CDF v18). Normalized gene expression estimates were obtained using the robust multi-array analysis (RMA) pre-processing algorithm available in the library 'AffyPLM' using default settings [5]. The dataset was filtered to only include probe sets that were active (i.e. expressed) in at least 4 samples using the universal expression code (UPC) approach (UPC score > 0.50) [6]. This resulted in the inclusion of 8,348 (39%) of the 21,266 probe sets. Differentially expressed probe sets were identified by using linear models and an intensity-based moderated t-statistic [7, 8]. Probe sets that satisfied the criterion of $P<0.05$ were considered to be significantly regulated. Array data have been submitted to the Gene Expression Omnibus under accession number GSE64104. Detailed information on microarray processing and data analysis is available upon request.

Biological interpretation of array data

Changes in gene expression were related to biologically meaningful changes using gene set enrichment analysis (GSEA) [9]. It is well accepted that GSEA has multiple advantages over analyses performed on the level of individual genes [9-11]. Gene sets were retrieved from

the expert-curated KEGG, Biocarta, Reactome and WikiPathways pathway databases. Only gene sets consisting of more than 15 and fewer than 500 genes were taken into account. Genes were ranked on their t-value that was calculated by the moderated t-test. Statistical significance of GSEA results was determined using 1,000 permutations. The Enrichment Map plugin for Cytoscape was used for visualization and interpretation of the GSEA results [12].

SUPPLEMENTAL REFERENCES

[1] Stienstra R, Duval C, Keshtkar S, van der Laak J, Kersten S, Muller M (2008) Peroxisome proliferator-activated receptor gamma activation promotes infiltration of alternatively activated macrophages into adipose tissue. J Biol Chem 283: 22620-22627

[2] Gentleman RC, Carey VJ, Bates DM, et al. (2004) Bioconductor: open software development for computational biology and bioinformatics. Genome biology 5: R80

[3] Lin K, Kools H, de Groot PJ, et al. (2011) MADMAX - Management and analysis database for multiple ~omics experiments. Journal of integrative bioinformatics 8: 160

[4] Dai M, Wang P, Boyd AD, et al. (2005) Evolving gene/transcript definitions significantly alter the interpretation of GeneChip data. Nucleic Acids Res 33: e175

[5] Irizarry RA, Bolstad BM, Collin F, Cope LM, Hobbs B, Speed TP (2003) Summaries of Affymetrix GeneChip probe level data. Nucleic Acids Res 31: e15

[6] Piccolo SR, Withers MR, Francis OE, Bild AH, Johnson WE (2013) Multiplatform single-sample estimates of transcriptional activation. Proc Natl Acad Sci U S A 110: 17778-17783

[7] Smyth GK (2004) Linear models and empirical bayes methods for assessing differential expression in microarray experiments. Stat Appl Genet Mol Biol 3: Article3

[8] Sartor MA, Tomlinson CR, Wesselkamper SC, Sivaganesan S, Leikauf GD, Medvedovic M (2006) Intensity-based hierarchical Bayes method improves testing for differentially expressed genes in microarray experiments. Bmc Bioinformatics 7: 538

[9] Subramanian A, Tamayo P, Mootha VK, et al. (2005) Gene set enrichment analysis: a knowledge-based approach for interpreting genome-wide expression profiles. Proc Natl Acad Sci U S A 102: 15545-15550

[10] Allison DB, Cui X, Page GP, Sabripour M (2006) Microarray data analysis: from disarray to consolidation and consensus. Nature reviews Genetics 7: 55-65

[11] Abatangelo L, Maglietta R, Distaso A, et al. (2009) Comparative study of gene set enrichment methods. Bmc Bioinformatics 10: 275

[12] Merico D, Isserlin R, Stueker O, Emili A, Bader GD (2010) Enrichment map: a network-based method for gene-set enrichment visualization and interpretation. Plos One 5: e13984

ESM TABLE 1: Descriptive characteristics of study type 2 diabetes patients (T2D) and controls (CON)

	T2D patients	Controls	P-value
N	45	72	
Age (yr)	60.3 ± 1.6	54.2 ± 1.0	0.0007 ***
Gender (female %)	44	54	
Duration diabetes (yr)	7.9 ± 0.9	-	
BMI (kg^2/m^2)	30.3 ± 0.8	27.6 ± 0.6	0.008**
WHR	1.02 ± 0.01	0.94 ± 0.01	<0.0001***
Fasting glucose (mmol/l)	11.6 ± 0.5	5.1 ± 0.1	<0.0001***
HbA1c (%)	8.7 ± 0.2	-	
TC (mmol/l)	4.2 ± 0.2	4.6 ±0.2	0.06
TG (mmol/l)	2.1 ± 0.2	1.2 ± 0.1	0.0002***
HDL (mmol/l)	1.1 ± 0.0	1.2 ± 0.1	0.06
LDL (mmol/l)	2.3 ± 0.2	3.0 ± 0.1	0.003**

Data are mean ± sem. BMI, body mass index; WHR, waist to hip ratio; HbA1c, glycated haemoglobin; TC, total cholesterol; TG, triacylglycerides; HDL high-density lipoprotein cholesterol; LDL low density lipoprotein cholesterol

ESM TABLE 2: Primers used for quantitative real-time PCR analysis

Gene	Forward primer	Reverse primer
Human		
B2M	ATGAGTATGCCTGCCGTGTG	CCAAATGCGGCATCTTCAAAC
SUCNR1	TGTGTCTAACACTGTTGGGGTTCC	TCCTCACATTCCGCATGACG
Mouse		
36B4	AGCGCGTCCTGGCATTGTGTGG	GGGCAGCAGTGGTGGCAGCAGC
Casp1	GGGACCCTCAAGTTTTGCC	GACGTGTACGAGTGGTTGTATT
Ccr1	TGGGTGAACGGTTCTGGAAG	GGTCCTTTCTAGTTGGTCCACA
Ccr5	ATGGATTTTCAAGGGTCAGTTCC	CTGAGCCGCAATTTGTTTCAC
Cd68	CCAATTCAGGGTGGAAGAAA	CTCGGGCTCTGATGTAGGTC
Cd74	AGATGCGGATGGCTACTCC	TCATGTTGCCGTACTTGGTAAC
Cd80	TGCTGCTGATTCGTCTTTCAC	GAGGAGAGTTGTAACGGCAAG
Cd86	GAGCTGGTAGTATTTTGGCAGG	GGCCCAGGTACTTGGCATT
Cxcr4	GACTGGCATAGTCGGCAATG	AGAAGGGGAGTGTGATGACAAA
Cyclophillin	TGTCTTTGGAACTTTGTCTGCAA	CAGACGCCACTGTCGCTTT
F4/80	CTTTGGCTATGGGCTTCCAGTC	GCAAGGAGGACAGAGTTTATCGTG
IL-1ra	AAATCTGCTGGGGACCCTAC	TGAGCTGGTTGTTTCTCAGG
iNOS	CGTTTCGGGATCTGAATGTGA	GGGCAGCCTGTGAGACCTT
Mrc1	CTCTGTTCAGCTATTGGACGC	CGGAATTTCTGGGATTCAGCTTC
Sucnr1	GGACCTATGGAGATGTTCTCTG	GGTAGAACTTCTAAGGTCACTAAG
Tnfa	CAGACCCTCACACTCAGATCATCT	CCTCCACTTGGTGGTTTGCTA

ESM TABLE 3: Pearson correlation coefficients between subject characteristics and plasma succinate levels in type 2 diabetes (T2D) patients and controls

**Variable**	**T2D patients (N=45)**		**Controls (N=72)**	
	R	*p-value*	R	*p-value*
Age (yr)	0.006	*0.96*	0.004	*0.98*
BMI	-0.065	*0.59*	-0.124	*0.42*
WHR	-0.049	*0.69*	-0.092	*0.55*
Fasting glucose (mmol/l)	0.176	*0.14*	0.165	*0.28*
HbA1c (%)	-0.06	*0.96*		
TC (mmol/l)	0.026	*0.84*	0.133	*0.38*
TG (mmol/l)	-0.182	*0.15*	-0.147	*0.34*
HDL (mmol/l)	-0.079	*0.53*	0.187	*0.22*
LDL (mmol/l)	0.126	*0.32*	0.110	*0.49*

Data are mean ± sem. BMI, body mass index; WHR, waist hip ratio; HbA1c, glycosylated haemoglobin, TC, total cholesterol, TG, triacylglycerides, HDL high-density lipoprotein cholesterol, LDL low density lipoprotein cholesterol

ESM Figure 1

A

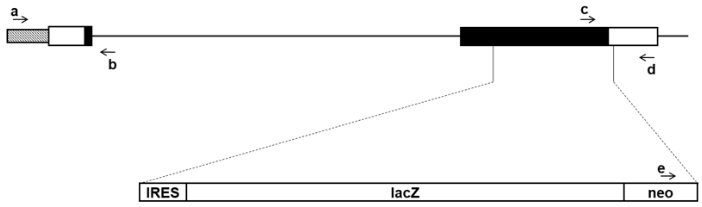

B

Primer	Sequence (5'→3')
a. Promoter	GCAAATCAGACTGCACTGGTC
b. Intron	GGCTGCTTCACATCTACTGAAG
c. Exon 2	GCTGTCTGGGCCTTAGTGACC
d. 3'UTR	GCTGCCTTCTGATTCATGTGG
e. Neomycin	GCAGCGCATCGCCTTCTATC

C

Primer combination	Expected band size (bp)
Promoter + Intron (a+b)	241
Exon 2 + 3' UTR (c+d)	701
Neomycin + 3' UTR (e+d)	333

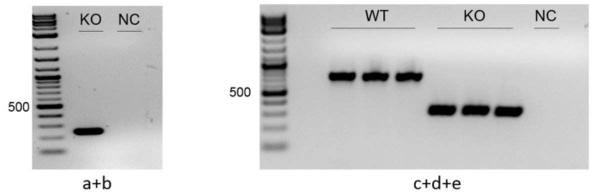

ESM Fig. 1 The SUCNR1 gene locus of the *Sucnr1*-knockout mouse

[A] Schematic map of the *gpr91/SUCNR1* wild-type (WT) locus: promoter (grey bar), untranslated regions (UTRs; white bars), intron (line) and coding sequence (CDS; black bars) are indicated. The IRES/lacZ/neo cassette was inserted to delete a large part of exon 2, by homologous recombination. Arrows and letters indicate primers used for confirmation of the genotype. [B] primers and their sequences used for genetic analysis. [C] Different combinations of primers (a+b and c+d+e) were used to establish the presence of exon 1 and the deletion of the CDS in *Sucnr1*$^{-/-}$ mice. Agarose gel electrophoresis of PCR results confirmed the presence of the expected bands. For each PCR reaction water was used as negative control (NC).

ESM Figure 2

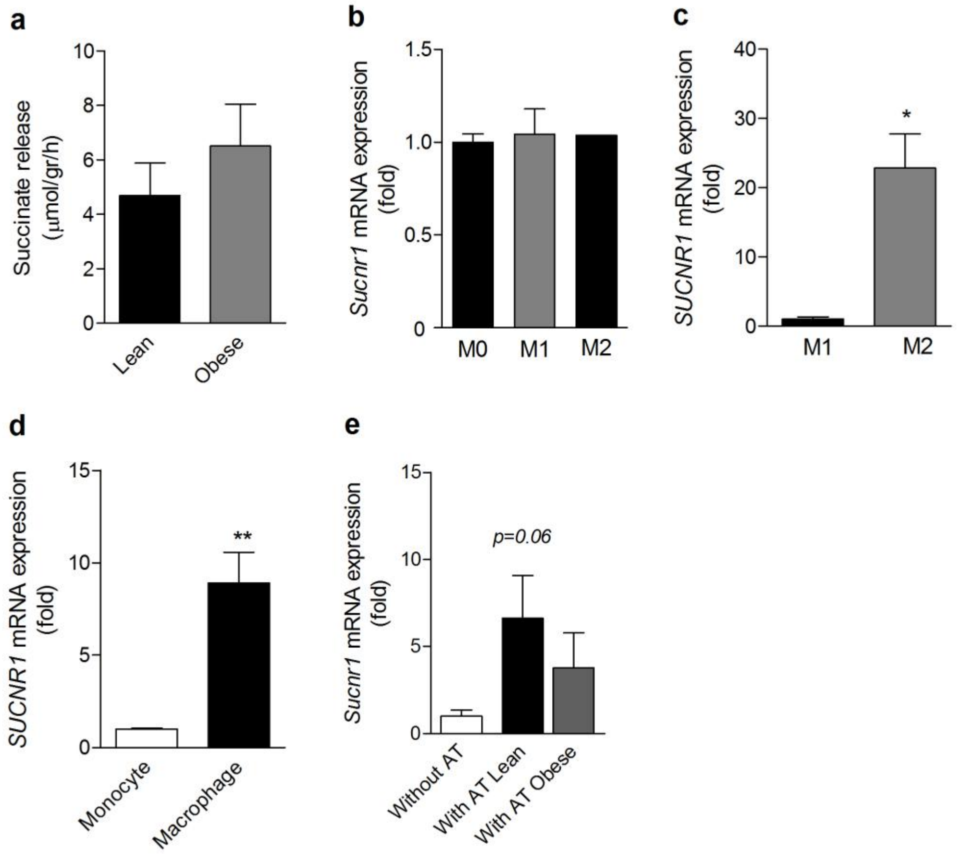

ESM Fig. 2. Succinate release from obese adipose tissue and SUCNR1 expression in murine and human macrophages

[a] Succinate release from mouse adipose tissue explants derived from obese versus lean mice. Explants were cultured *ex vivo* in 1mmol/l glucose medium. [b-e] *SUCNR1* mRNA expression was evaluated in publically available microarray data sets comparing M1 versus M2 macrophages from human (GSE5099) and mouse (GSE69607) origin. [b] Mouse bone marrow derived M0 macrophages (medium control); M1 macrophages (LPS + IFNγ stimulated, 100ng/mL and 20ng/mL respectively) and M2 macrophages (IL-4 stimulated, 20 ng/mL) (n=2-3). [c] Human M1 macrophages (LPS + IFNγ stimulated, 100ng/mL and 20ng/mL respectively) versus M2 macrophages (IL-4 stimulated, 20 ng/mL) [d] Human monocytes and differentiated macrophages using autologous serum (n=3). [e] Mouse bone marrow derived macrophages exposed to adipose tissue (AT) explants from lean and obese mice. Data are fold change as compared to control. Data from [e] are tested using a 1-way ANOVA Kruskal-Wallis test. Data are mean ± SEM from n=4 replicates. *p<0.05, ** P<0.01.

ESM Figure 3

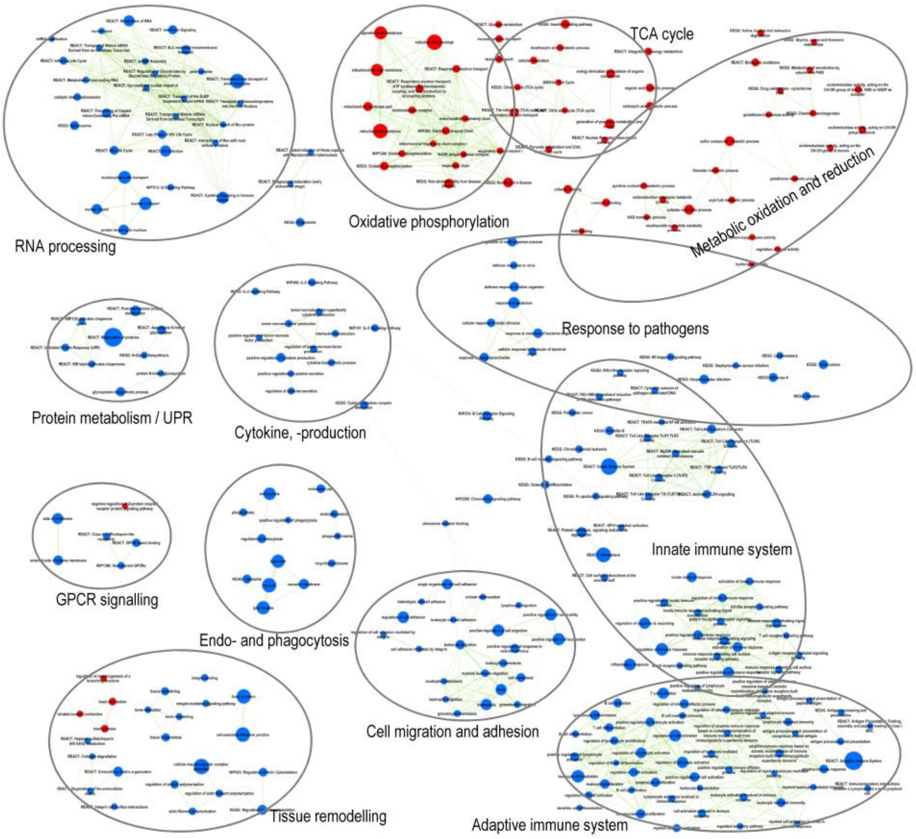

ESM Fig. 3. Absence of SUCNR1 reduces inflammatory pathways within adipose tissue Enrichment map showing differentially-regulated pathways in adipose tissue of *Sucnr1*$^{-/-}$ as compared to wildtype (WT) mice. GSEA was performed to identify functional gene sets, i.e. metabolic pathways or signaling transduction routes, that were changed in *Sucnr1*$^{-/-}$ mice ($p<0.001$, FDR<0.25). Nodes represent gene sets, and edges between nodes represent their similarity. A red node indicates induction of a gene set, and a blue node indicates suppression of a gene set in *Sucnr1*$^{-/-}$ compared to WT. Node size represents the gene set size, and edge thickness represents the degree of overlap between 2 connected gene sets. Gene sets were grouped by cluster analysis, applying the Markov Cluster Algorithm, which were semi-automatically annotated and manually labeled to highlight the prevalent biologic functions among the related gene sets.

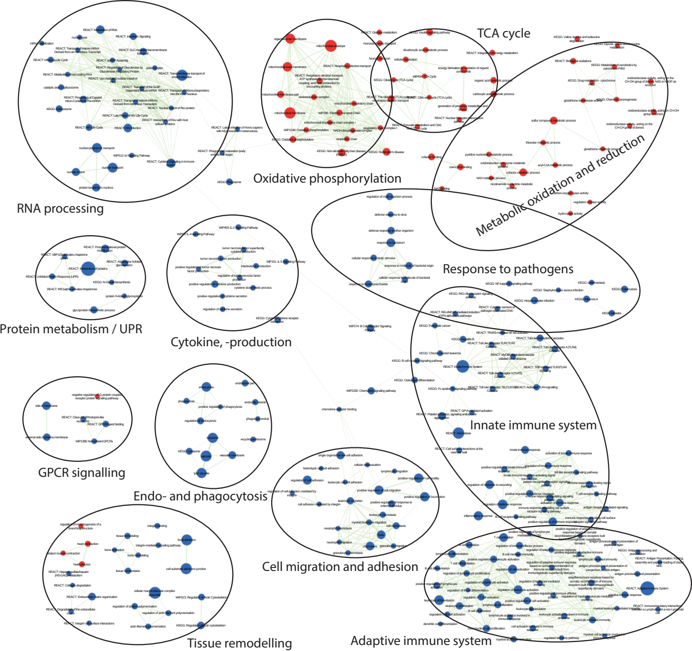
RNA processing
Oxidative phosphorylation
TCA cycle
Metabolic oxidation and reduction
Response to pathogens
Protein metabolism / UPR
Cytokine, -production
Innate immune system
GPCR signalling
Endo- and phagocytosis
Cell migration and adhesion
Tissue remodelling
Adaptive immune system

ESM Figure 4

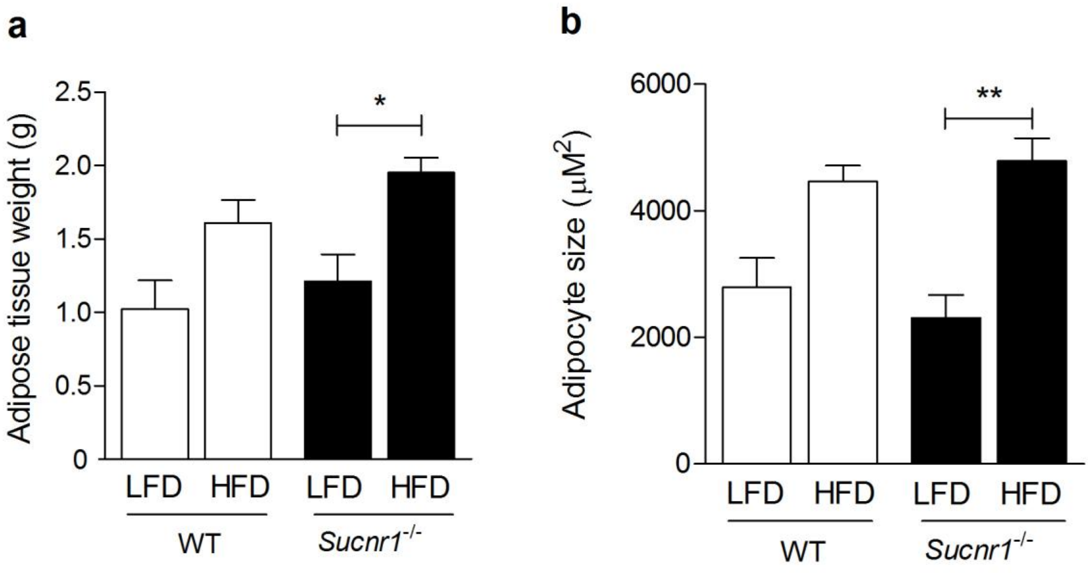

ESM Fig. 4. Absence of SUCNR1 does not affect adipose tissue weight or adipocyte size.

Sucnr1$^{-/-}$ and wildtype (WT) mice were subjected to a LFD or HFD feeding for 16 weeks. [a] Adipose tissue weight after 16 weeks of LFD or HFD feeding. [b] Average adipocyte size distribution of epididymal adipose tissue. Data are mean ± SEM from n=7 animals per group. *p<0.05, ** P<0.01.

ESM Figure 5

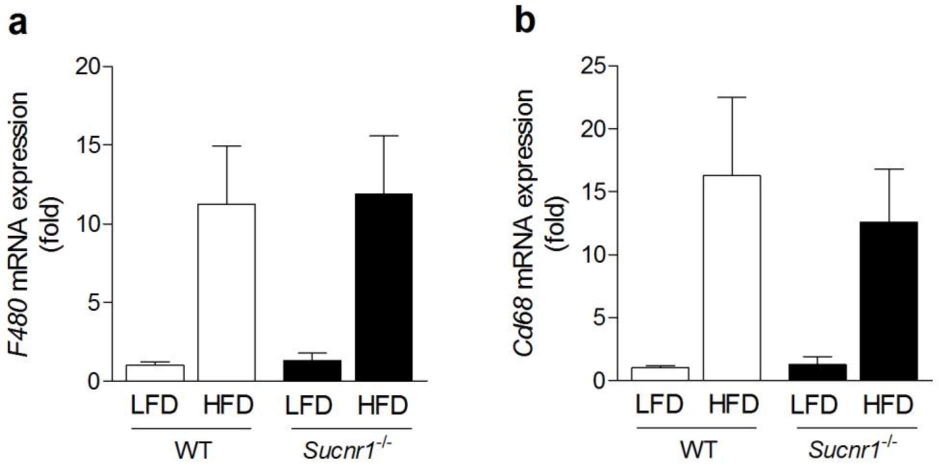

ESM Fig. 5. Absence of SUCNR1 does not affect macrophage infiltration after 8 weeks of high fat diet (HFD) feeding. Relative mRNA levels of [a] *F4/80* and [b] *Cd68* in epididymal white adipose tissue of *Sucnr1*$^{-/-}$ and wild type (WT) mice after low fat diet (LFD) or high fat diet (HFD) feeding for 8 weeks. mRNA levels of WT mice fed a LFD are set to 1. Data are fold change as compared to WT LFD. Data are mean ± SEM from n=5-7 animals per group.

ESM FIGURE 6

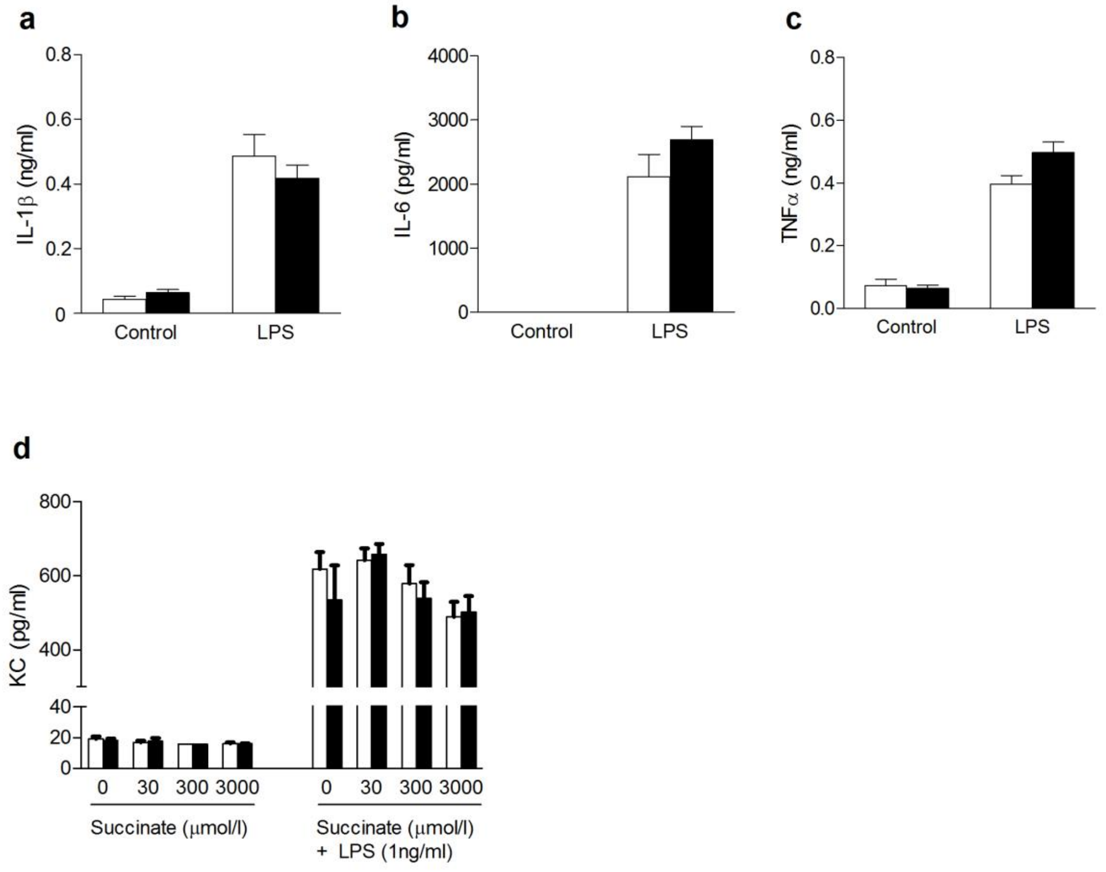

ESM Fig. 6. Absence of SUCNR1 does not affect macrophage cytokine or chemokine secretion.

Peritoneal cells were isolated from *Sucnr1*$^{-/-}$ (black bars) and wildtype (WT) mice (white bars) and incubated with or without LPS (10 ng/ml) for 24h. Intracellular levels of IL-1β [a] as well as secretion of IL-6 [b] and TNFα [c] were deteremined. Bone-marrow derived macrophages were incubated with succinate with or without LPS (1ng/ml) and levels of KC were determined [d]. Data are mean ± SEM; n=4-7 mice.

ESM Figure 7

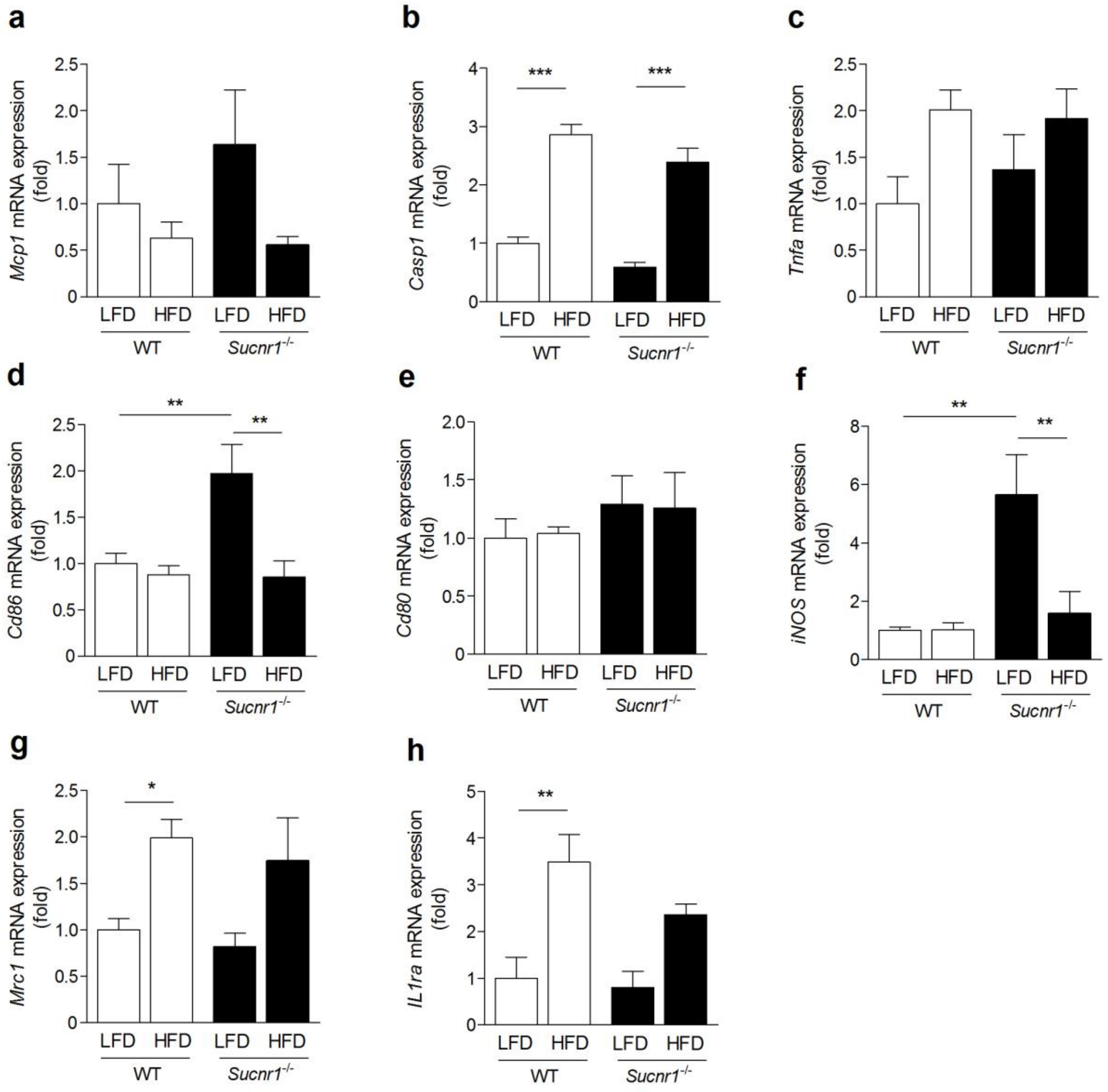

ESM Fig. 7. Absence of SUCNR1 does not affect (anti)-inflammatory macrophage phenotype after high fat diet (HFD) feeding. Relative mRNA levels of [a] *Caspase-1*, [b] *TNFα*, [c] *Cd86*, [d] *Cd8*0, [e] *iNOS*, [f] *Mrc1*, [g] *IL-1ra* in epididymal white adipose tissue of *Sucnr1*$^{-/-}$ and wild type (WT) mice after low fat diet (LFD) or high fat diet (HFD) feeding for 16 weeks. Data are fold change as compared to WT LFD. Data are mean ± SEM from n=6-7 animals per group. *p<0.05, ** P<0.01, *** P<0.001

ESM Figure 8

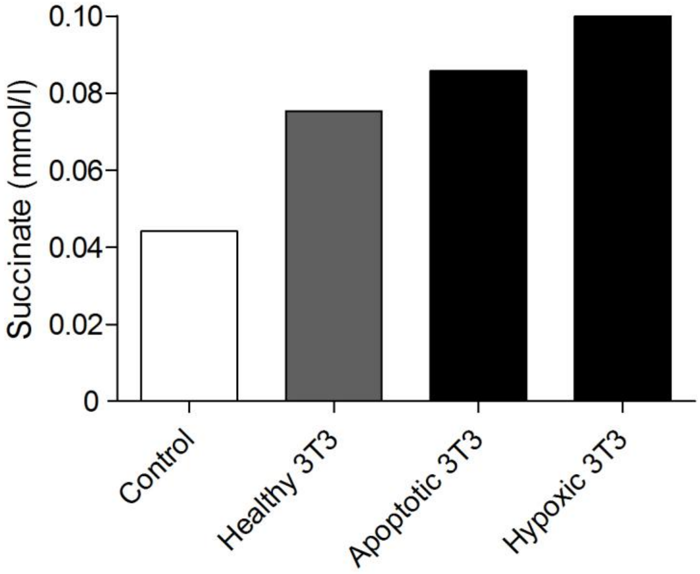

ESM Figure 8. Succinate is released from apoptotic and hypoxic 3T3 adipocytes. Pooled medium was used for determination of succinate using 1H NMR spectroscopy. Mean succinate levels are shown for control medium (DMEM/10% serum/1%pen/strep), medium derived from healthy 3T3 adipocytes, apoptotic adipocytes and hypoxic adipocytes.

ESM Figure 9

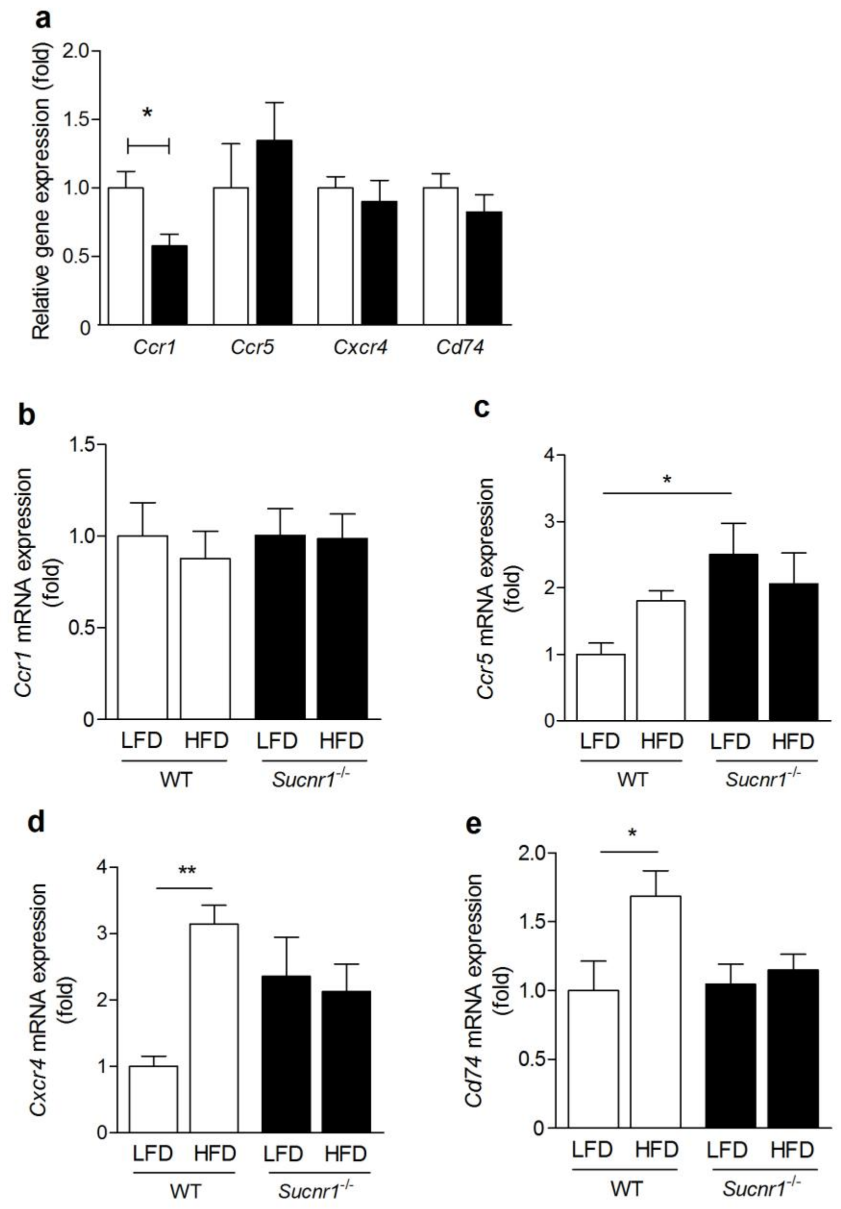

ESM Figure 9. Expression of chemokines receptors in BMDMs and adipose tissue of *Sucnr1*$^{-/-}$ and wild type (WT) mice. [a] Relative mRNA levels of *Ccr1, Ccr5, Cxcr4* and *Cd74* in bone marrow derived macrophages (BMDMs) of *Sucnr1*$^{-/-}$ and wild type (WT) mice. Relative mRNA levels of [b] *Ccr1,* [c] *Ccr5,* [d] *Cxcr4*, [e] *Cd74* in epididymal white adipose tissue of *Sucnr1*$^{-/-}$ and wild type (WT) mice after low fat diet (LFD) or high fat diet (HFD) feeding for 16 weeks. Data are fold change as compared to WT LFD. Data are mean ± SEM from n=6-7 animals per group.
